# Sixth Annual Meeting of the American Society of Dental Surgeons

**Published:** 1845-09

**Authors:** 


### Miscellaneous Notices.

### SIXTH ANNUAL MEETING OF THE AMERICAN SOCIETY OF DENTAL SURGEONS.

The Society met at the Stuyvesant Institute, Tuesday, August 5th, 1845, at 11, A. M.

The roll was called, and the following were the members present: E. Parmly, Lewis Roper, Solyman Brown, C. A. Harris, J. H. Foster, John Allen, E. Baker, G. G. Brewster, M. K. Bridges, H. S. Burr, J. W. Cowan, E. J. Dunning, John Harris, S. P. Hullihen, O. P. Laird, John Lovejoy, Alex. Nelson, Robert Nelson, Enoch Noyes, Jahial Parmly, S. W. Parmly, S. W. Stockton, James Taylor, J. Taylor, E. Townsand, A. Westcott, H. H. Young, L. S. Allen, John M. Howe, John B. Rich.

The minutes of the preceding year were read and approved.

The following were appointed a committee to arrange the business of the meeting, viz. Drs. E. Baker, E. Townsand and A. Westcott. After a short conference, they recommended the following order:

*First.*—To receive the reports of committees, viz.

1. To prepare a memorial to present to the Legislature, in behalf of the American Society of Dental Surgeons.
2. The Treasurer's Report.

*Second.*—Addresses and Essays.

1. Opening Remarks, by E. Parmly.
2. Opening Address, by J. H. Foster.
3. Essay, by E. Townsand.
4. Essay, by E. J. Dunning.
5. Essay, by J. Taylor.

*Third.*—Voluntary Essays.

1. Essay, by E. Baker.
2. Essay, by J. Allen.

*Fourth.*—Promiscuous Business.

A letter from Dr. C. A. Harris was then read, and action upon it was deferred till it was certain whether he would be present at the meeting.

On motion of Dr. J. B. Rich, the following resolution was offered and adopted, viz.

*Resolved,* That a committee of investigation be appointed; such committee to consist of five members of this association, to be nominated by the chair. The duty of the committee, so appointed, shall be to call upon each of the members *now* in this city, with the view to ascertain from each member whether he has used any amalgam in the course of his own practice as a dental surgeon, or approves of its use; and if he has used it, whether he has done so within the last twelve months.

*And it is further Resolved,* That the said committee of five be particularly requested to obtain, if possible, a direct answer on this subject, from each and every member so called upon.

The committee appointed pursuant to the above resolution, was the following, viz. Drs. J. B. Rich, J. Taylor, J. Allen, E. J. Dunning, and Alex. Nelson.

After the passage of the above resolutions, a spirited debate ensued, not only in relation to the rights of the Society, so to call upon each member, and to demand an exposition of his practice, but also in relation to the propriety of ever using it, under any circumstances. Among those who advocated the ground of this right, and that its use was *never* admissible, were E. Parmly, J. Parmly, J. B. Rich, A. Westcott, A. Nelson, and several others.

The principal advocate for the position that it was occasionally admissible, was E. Baker. Although Dr. B. admitted that it was, in general terms, a "vile and *nasty* substance, and that it was capable of producing only the worst of effects, unless when used in teeth which could not be filled in any other way," he contended that, in "certain cases," it could be judiciously employed. Dr. S. Brown questioned the right of the Society to demand of its members their private practice, and contended that "no one ought to be required to pledge himself not to use any article, as his *conscience* might demand that he should use it."

Adjourned to Wednesday, 9, A. M.

*Wednesday, August* 6th, 9, A. M.

After the calling of the roll, an address was given by the President, Dr. E. Parmly.

On motion of Dr. C. A. Harris,

*Resolved,* That the address of Dr. P. be requested for publication, and that a committee be appointed to take into consideration certain parts of it, with reference to carrying out many important suggestions which it contained.

Drs. C. A. Harris, Westcott, S. Brown, Bridges, were that committee.

After this, Dr. J. H. Foster gave his opening address, which was ordered to be published, subject to the revision of the above committee, in connection with Dr. Foster.

Dr. E. Townsand read an essay, which was ordered to be published.

Dr. Harris read a letter from W. A. Palmer, asking the Society if it would grant him a diploma, on certain compromising terms.

On motion of Dr. J. B. Rich,

*Resolved,* That his letter be laid upon the table; that his name be stricken from the list of members, and that he be notified of the action of the Society upon his case.

A letter was read from J. Smith Dodge, tendering his resignation.

On motion of J. B. Rich,

*Resolved,* That his resignation be not accepted.

Adjourned to 4½, P. M.

Met agreeably to adjournment, at 4½, P. M.

The meeting was opened by an essay from E. J. Dunning.

Mr. D's essay was ordered to be printed in the Journal.

Dr. James Taylor read an essay, which was also requested for publication.

Adjourned to 9, to-morrow, at this place.

*Thursday,* 9, A. M.

Dr. E. Baker read an essay, which was requested for publication. Whereupon S. P. Hullihen offered his "protest" to its publication without stricture; and Dr. H. proceeded to comment on certain portions of it. This, however, was objected to, as being out of order, and the essay ordered to be published.

The committee formed for the purpose of calling upon the different members of the Society, to get information relative to their practice in respect to using mineral paste for plugging carious teeth, made the following Report, viz.

No. 2. *Report of the Committee of Investigation.*

The committee of investigation, nominated by the chair, and deputed to call upon the several members of this Society at present in the city of New York, for the purpose of ascertaining the views of each member as it regards the use of amalgam in dental surgery, report,

That there are twenty-five members of this Society resident in the cities of New York and Brooklyn. Of this number two were absent, to wit: J. Smith Dodge, and Wm. Arnold. The committee, therefore, could not learn the opinions of these gentlemen, touching the use of amalgam.

The committee did not deem it advisable or necessary to call upon I. I. Greenwood or David Rossiter, forasmuch as these gentlemen have not practised during the last twelve months.

On the remaining twenty-one members, the committee of five waited, and ascertained that ten of them disapprove entirely of the use of amalgam, and declare that they have never used it in their practice. The names of these ten members are as follows: Eleazar Parmly, Jas. Alcock, J. H. Foster, Elihu Blake, Jahial Parmly, C. S. Rowell, P. Houston, Sam'l W. Parmly, E. J. Dunning, John B. Rich.

Of the eleven members still remaining, the committee have ascertained that five have used amalgam in certain cases; but these five are willing to pledge themselves to abandon altogether its use for the future. Here follow the names of the five members who have so pledged themselves: Elisha Baker, M. K. Bridges, Benjamin Lord, Chas. O. Baker, J. M. How.

Six use amalgam under certain circumstances, and refuse to pledge themselves to discontinue its use. The names of these six are: John Lovejoy, Nehemiah Dodge, A. W. Brown, George E. Hawes, C. C. Allen, F. H. Clark.

With respect to the non-resident members of this body, now sojourning in the city of New York, the committee have called upon no less than twenty-one. The whole disapprove, unqualifiedly, of the use of amalgam, and only one has used it within the last twelve months, and then merely by way of experiment. These are the names of the twenty-one: Chapin A. Harris, Amos Westcott, J. Taylor, E. Townsand, S. Stockton, Solyman Brown, A. Hill, Alex. Nelson, H. H. Young, John Harris, G. G. Brewster, D. D. Crispin, Charles Walker, R. Nelson, O. P. Laird, E. Noyes, J. Allen, S. P. Hullihen, S. G. Pancost, H. S. Burr, Edw. Jamet.

Respectfully submitted,

JOHN B. RICH,

M. K. BRIDGES,

GEO. G. BREWSTER, } *Committee.*

E. NOYES,

E. J. DUNNING,

This report was accepted.

Previous to its acceptance, several gentlemen spoke at some length, in respect to the very pernicious effects of this article, and of the painful reflection in consequence of its having been used by some of our members, even after they knew it to have been thrice declared, by strong resolutions of this association, to be malpractice. There was a unanimous wish expressed, that the action upon it should be efficient, positive and final. Whereupon Dr. J. B. Rich offered the following resolution:

*Resolved,* That the report just received be referred to a committee of five, to be appointed by the chair. The committee to report immediately some plan of action for this Society to take in this matter.

The following were this committee, viz. Drs. Hullihen, J. Taylor, E. Noyes, Rich, C. A. Harris.

A committee was then appointed to examine the Treasurer's report. That committee was: Drs. Bridges, E. Townsand, and E. J. Dunning.

After a short conference, they presented the following report, which was unanimously adopted by the Society, viz.

The committee appointed to examine and audit the account of the Treasurer, respectfully report, that they have performed this duty, and find it highly satisfactory, and is as follows:

| | |
|---|---|
| Rec'd from members and subscribers, during the past year, | $ 1486 87 |
| Paid expenses of Journal, &c. . . . . . . . | 1251 35 |
| Due for membership, . . . . . . . . . | 1600 00 |
| Due for Journal, . . . . . . . . . . | 1360 00 |

They beg leave, in addition, to state, that, on account of the disordered state of the accounts at the time they were submitted to Dr. Harris, he has had uncommon labor in their adjustment; also, an actual outlay of $ 50 00 to an assistant, to bring them into their present state. In addition to this, he has been obliged, in consequence of the loss of eighteen half volumes of the Journal, to break his own set, that those for whom they were intended in Europe might not be disappointed. Your committee would respectfully suggest that this Society refund to Dr. Harris the $ 50 actually expended by him, and fully reimburse him for the loss sustained in the eighteen volumes of the Journal; and also to request his acceptance of $ 100, as some small expression of our sense of the desirable result produced by his labors. They would further suggest that the thanks of the Society be most cordially given to Dr. Harris, for the very efficient manner in which he has brought chaos into order."

Adjourned till 5, P. M.

Met at 5, P. M.

Dr. John Harris read his essay, which was requested for publication.

On motion of C. A. Harris,

*Resolved,* That Dr. Robert Arthur have his dues remitted, and that he be furnished with the Journal for the term of seven years, from the time his membership commenced, for his services in translating Blandin's work.

A letter of resignation was read, from Drs. E. G. and J. Tucker, of Boston, by Dr. Foster, who offered some very appropriate suggestions in relation to it. The chair appointed Dr. Foster a committee to confer with the Messrs. Tucker, and, meanwhile, any action was suspended by the Society upon said letter.

A letter was read from Dr. Maynard, preferring charges of unprofessional conduct against Dr. J. Smith Dodge, and requesting some action of the Society upon them.

By request of the President, Dr. E. Parmly, Dr. E. Townsand, Vice-president, was called to the chair, pro tem.

On motion,

*Resolved,* That a committee be appointed to make some report upon the charges contained in Dr. Maynard's letter.

This committee was appointed by the chair, and was as follows: Drs. J. B. Rich, J. H. Foster, H. H. Young.

This committee found it necessary to delay their report, as Dr. Dodge could not be seen, and they deeming it proper that his defence, if any, should be heard. The subject, therefore, is left in the hands of the committee, to be presented at a proper time.

On motion of Dr. E. Parmly, a committee was formed to draft a letter, addressed to editors of journals, newspapers, &c., upon the subject of quackery. This committee is as follows: Drs. E. Parmly, C. A. Harris, S. Brown, J. H. Foster, A. Westcott. They were requested to meet at the Atheneum Hotel.

Dr. Harris read a letter of resignation of membership from E. G. Kelly.

Also, one from Elbridge Bacon, in which he declared himself not to be a member of the Society, and declined paying his dues. This letter was voted to be laid upon the table, on the ground that it did not come within the recognition of the Society, Mr. Bacon having already forfeited his membership.

Adjourned to 9, to-morrow.

*Friday*, 9, A. M.

Dr. J. Allen, of Cincinnati, read an essay, and exhibited an apparatus for restoring the shape of the face, lost from any cause.

On motion of Dr. Brewster,

*Resolved*, That Dr. Allen's improvement be regarded as important, and that a medal be presented him, also five volumes of the Journal, bound and subscribed by the Recording Secretary, as awarded by the Society for said improvement.

Dr. Bridges introduced the subject of taking impressions with plaster. Several gentlemen offered remarks upon the subject, &c.

The committee to whom was referred Dr. E. Parmly's address, made the following report, viz.

1. They recommend the appointment of a committee, to prepare a set of aphorisms, on the most important subjects of dental practice.

2. A committee of ten, to whom these aphorisms shall be submitted for review, and, if necessary, revision and sanction, previous to publication.

3. That these aphorisms shall not fill more than twelve duodecimo pages.

4. That twenty-five dollars be appropriated by the Society for their publication.

Adopted.

The following are the subjects selected, together with the individual appointed to write upon each, respectively, viz:

| | |
|---|---|
| 1. Qualifications of dentists, | Amos Westcott. |
| 2. Filling teeth, | E. Parmly. |
| 3. Filling teeth, | E. Townsand. |
| 4. Cleansing, | E. J. Dunning. |
| 5. Regulating, | L. Roper. |
| 6. Toothache, | J. Harris. |
| 7. Artificial teeth, | S. Brown. |
| 8. Morbid effects of diseased teeth, | C. A. Harris. |
| 9. Attention, teeth require, | Wm. Dwinelle. |
| 10. Diseases of the gums, | E. Baker. |
| 11. Extracting teeth, | M. K. Bridges. |
| 12. Tooth washes and powders, | H. S. Burr. |
| 13. Amalgams for filling, | J. H. Foster. |

The following were appointed as the reviewing committee: Noyes, Young, C. S. Allen, O. Holmes, McIlhany, A. Nelson, J. Alcock, Brewster, Houston, and Stockton.

Lastly, these subjects are to be revised by the President and both Secretaries.

The committee, who were appointed to report some plan of action in relation to mineral paste, submitted the following, viz.

"The committee who were directed to suggest some mode or plan of action for the adoption of this Society, with respect to the use of amalgams in dental practice, and to whom was referred the report of the visiting committee of investigation, report—

"That they have deliberated carefully upon the matter referred to them, and that their unanimous opinion is, that any amalgam is not only unfit, but dangerous, when used for the purpose of filling carious teeth or their fangs. That it is, therefore, the imperative duty of the American Society of Dental Surgeons to express, most distinctly and unqualifiedly, their disapproval of its use for the above named purpose under any circumstances.

The committee, therefore, submit for adoption, the following resolutions, viz.

*Resolved,* That the American Society of Dental Surgeons, under the conviction that any amalgam whatever, whether used under the name of "mineral paste," "adamantine cement," "succedaneum," "diamond cement," "lithodeon," "alabaster cement," "Chinese cement," or in any other way designated, is not only unfit, but dangerous, when used for filling the teeth or their fangs, do hereby pronounce the use of all amalgams as malpractice.

*And it is furthermore Resolved,* That any member of this Society who shall hereafter refuse to sign a certificate pledging himself not to use any amalgam, and, moreover, protesting against its use, under any circumstances, in dental practice, shall be expelled from this Society.

Signed,

S. P. HULLIHEN,
J. TAYLOR,
E. NOYES,
C. A. HARRIS,
J. B. RICH.

On motion, the report was accepted.

On motion of J. B. Rich, the resolutions were adopted.

On motion of Dr. A. Nelson, the necessity of adopting some amendments to the above resolutions was urged, on the ground that they were too indefinite—that, although they provided for the expulsion of members who should use this article, yet they did not point out any definite time. It was urged, further, that if the time for the signing of the certificate, which was provided by the above resolutions, was to be confined to the session of that particular meeting, it might do injustice to those out of the city, and particularly those out of the country. With the view, therefore, to make the matter definite; and, also, that equal and exact justice might be done to all, Dr. J. B. Rich offered the following resolution as an amendment to the foregoing, viz.

*Resolved,* That the Recording Secretary be, and is hereby, directed to forward to every member of this Society, within thirty days after the 10th day of August, 1845, a printed copy of the resolutions passed at this convention of the American Society of Dental Surgeons, relative to the use of amalgams under any form or name.

*Resolved, further,* That the Recording Secretary be requested, at the same time, to transmit to the several members, as aforesaid, a printed protest or certificate, in accordance with the above-mentioned resolutions, notifying each member that unless he shall subscribe with his own proper signature the said protest or certificate, and return the same to the Recording Secretary within sixty days from the time the said notice is issued, or, in the case of those living out of the United States, within one year from the first day of August, 1845, he shall be considered as ineligible to be a member of the American Society of Dental Surgeons, and shall stand expelled from the same.

*And it is furthermore Resolved,* That the Recording Secretary be, and he is hereby, directed, without further reference, to strike from the roll of this Society, after the expiration of the above specified terms, the names of such members as may have failed to comply with the terms and conditions set forth in the foregoing resolutions.

Unanimously adopted.

The report relative to J. Smith Dodge was again brought up, and again deferred, with the hope that Dr. D. would be present to answer to the charges, he having been twice notified that his case was before the Society for trial.

The committee appointed to devise and report some plan for getting legislative action regulating the practice of dentistry in this state, recommended that a committee be appointed by ballot, to carry out the wishes of the Society upon this subject.

The committee was as follows:

Drs. Fenn, Rochester; Young, Troy; Alexander Nelson, Albany; S. Brown, Ithaca; E. Parmly, New York; Westcott, Syracuse; Smith, Binghampton, N. Y.; Bridges, Brooklyn; Dwinelle, Cazenovia, N. Y.

On motion of Dr. Rich,

*Resolved,* That a committee of *five,* a majority of whom shall reside in one city, or so convenient to each other as that they may meet often for the purpose of deliberation, be, and hereby is appointed, to revise, alter, amend and add to the constitution and by-laws of this the American Society of Dental Surgeons; and that it shall be the duty of such committee to report progress at the commencement of the annual convention to be held in the month of August, 1846.

*Resolved, also,* That the report of said committee, and the consideration of any alterations, amendments, and additions, that may have been suggested by said committee of five, shall be the first business in order, after the delivering of the opening address.

*Resolved, also,* That during the present convention of this the American Society of Dental Surgeons, to wit: the session of August, 1845, there shall be no election for the admission of any new members into this association.

*Resolved, also,* That all candidates for admission, who may be proposed at the present session, can only be admitted according to the requirements of the constitution and by-laws of the Society at the time they are balloted for.

*Resolved, furthermore,* That such candidates shall be apprised of the nature and requirements of the constitution and by-laws, previous to the balloting, and have permission to withdraw their application should it be their wish so to do.

Adopted.

The following gentlemen were appointed as the committee: J. B. Rich, Lewis Roper, C. A. Harris, J. Parmly, J. H. Foster.

On motion of S. W. Stockton,

*Resolved,* That the Secretaries' dues be remitted, in consequence of extraordinary duties.

Carried.

Adjourned to 4, P. M.

Met, agreeably to adjournment, at 4, P. M.

Dr. Westcott offered the following resolution, which was adopted by the Society.

*Resolved,* That this society view the publication, by dentists, in connection with their advertisements, of letters of recommendation from divines, doctors of medicine, and, in short, all who are not well acquainted with dental practice, with decided disapprobation; and they would specially recommend to all its members, who may be pursuing this course, to discontinue a practice savoring so much of quackery, and one so well calculated to degrade the profession.

On motion of J. B. Rich,

*Resolved,* That each member be authorised by this Society to publish, in any newspaper, the above resolution at his own expense.

The committee appointed to prepare a letter, to be addressed to editors of newspapers, journals, &c., reported the following, which was adopted by the Society, viz.

"*New York, August* 9, 1845.

"To the editors of newspapers, magazines, and other periodicals in the United States, from the American Society of Dental Surgeons, at its sixth annual meeting, held in the hall of the Medical Department of the New York University, August 5th, 6th, 7th and 8th, 1845.

"Gentlemen—The objects of this Society are the mutual improvement of its members and the protection of themselves and the public against the quackery and empiricism which are the disgrace of the profession.

"The Society does not presume, in this communication, to speak of more than a single one of those base deceptions by which individuals calling themselves dentists are grossly imposing on the community. We allude to the practice of stopping decayed teeth with amalgams, known under the names of royal succedaneum, lithodeon, mineral paste, adamantine cement, alabaster cement, diamond cement, and other improper substances, by the use of which, thousands of valuable teeth are annually destroyed, and immeasurable evils result to the community at large which can never be repaired.

"The Society has declared by unanimous resolution that the use of the above named amalgams for stopping teeth is malpractice, destructive to the safety of the teeth, injurious to the healthy condition of the mouth, and not unfrequently exciting and promoting bad effects on constitutions frequently disposed to the injurious action of mercury, which invariably constitutes an ingredient in all these compounds. Every member of this Society who shall hereafter use this substance under any of these imposing and deceptive names, or under any other name, is, by that act, expelled from the institution.

"By order of the Society.

E. PARMLY, *President.*

AMOS WESTCOTT, *Recording Secretary.*"

On motion of E. J. Dunning, the following resolution, in relation to receiving application for membership, was adopted.

*Resolved,* That this Society, at its present session, shall receive all applications, duly made for membership, which shall be presented.

*Also, Resolved,* That the Corresponding Secretary, after having received a list of such applicants from the Recording Secretary, shall inform each applicant of the reasons which have led the Society to postpone the admission of any member, until the next session.

On motion of Dr. Bridges,

*Resolved,* That that part of Dr. Elliott's letter to Dr. Harris, relative to a case of instruments kindly sent to the Society by Dr. Elliott, for public exhibition, and the inspection of its members, at the discretion of the editors, be printed in the next number of the Journal.

*Resolved, further,* That the hearty thanks of the members of the American Society of Dental Surgeons, now assembled, be tendered through the Journal to Dr. Elliott for the very great liberality and generosity manifested on his part in thus publicly exhibiting to them the beautiful specimens of his inventive genius and achievements in the production of the instruments now before them.

A committee was appointed to report to the Society, who stood expelled, by the requirements of the constitution and by-laws, and who, by charges preferred against them.

That committee was Drs. C. A. Harris and Solyman Brown.

Dr. Brown was appointed as special agent of the committee appointed to carry out the wishes of the Society in relation to legislative action.

Adjourned to 8, P. M., to E. Parmly's, No. 1 Bond street.

Met agreeably to adjournment at 8, P. M.

Dr. Harris, on the above committee to report to the society the standing of members, read the names of those who had been elected, by friends, without their knowledge or consent, and whose names were, for this reason to be stricken from the list. They were, as follows, viz. Drs. Chester Hayden, William Arnold, E. Bacon, C. O. Baker, E. Blake, —— Blakesly, S. Blandin, W. H. Burr, N. Clute, J. W. Cowen, David Rossiter, —— Sumner, E. Hudson, Jahial Parmly, of Savannah; R. Somerby, W. A. Ward, H. B. Lathrop, S. Spooner, John G. Wayte, —— Macklin, B. A. Rodrigues, S. B. Straw and P. N. Williams.

The following are the names of the members decided to be expelled by the requirements of the constitution and by-laws, viz. J. O. Baldwin, F. H. Clark, V. Cuyler, —— Foster of Utica, and A. C. Hawes.

A list of charges was brought by Wm. G. Lord against J. O. Baldwin, of Newark, N. J., and said Baldwin was voted to be immediately expelled from the Society.

On motion of Dr. Brown,

*Resolved,* That the Recording Secretary be requested to forward to "the Forceps," the action of this Society upon said Baldwin's case, for publication.

*Resolved, further,* That Dr. Lord be authorised to publish the above resolution in the public papers of Newark, and that a copy be furnished him by the Recording Secretary.

On motion of Dr. J. H. Foster,

*Resolved,* That the thanks of the Society be presented to Dr. Stockton for publishing for the Society.

On motion of Dr. Harris,

*Resolved,* That Dr. Stockton's advertisement be published in the Journal the same length of time as he has published for the Society.

On motion of Dr. Brown,

*Resolved,* That each editor receive fifty dollars, and five copies of the Journal.

On motion of Dr. Foster,

*Resolved,* That the Society be a vigilance committee, to report to it all cases of malpractice which may come to the knowledge of any of its members.

On motion of Dr. J. Allen,

*Resolved,* That a synopsis of the minutes of the meeting be furnished for publication in newspapers.

Dr. S. Brown was chosen to perform the task.

Dr. J. H. Foster was voted to be furnished with a copy of the Journal from the beginning for his services as librarian.

The Society now proceeded to the election of officers.

The balloting resulted as follows:

| | |
|---|---|
| E. PARMLY, | PRESIDENT. |
| N. C. KEEP, | 1ST VICE-PRESIDENT. |
| LEWIS ROPER, | 2D VICE-PRESIDENT. |
| JOHN A. CLEAVELAND, | 3D VICE-PRESIDENT. |
| C. A. HARRIS, | CORRESPONDING SECRETARY. |
| AMOS WESTCOTT, | RECORDING SECRETARY. |
| C. A. HARRIS, | TREASURER. |
| J. H. FOSTER, | LIBRARIAN. |

*Editors of the American Journal and Library of Dental Science.*

C. A. HARRIS, A. WESTCOTT, E. MAYNARD.

*Executive and Examining Committee.*

E. BAKER, J. B. RICH, E. TOWNSAND,

J. H. FOSTER, J. PARMLY, E. J. DUNNING,

J. LOVEJOY.

*Publishing Committee.*

E. TOWNSAND, L. ROPER, H. S. BURR.

Opening address, Dr. Bridges.
First Essay, Dr. Rich.
Second Essay, C. C. Allen.
Third Essay, Wm. H. Dwinelle.
Fourth Essay, W. H. Elliott.
Fifth Essay, G. G. Brewster.

On motion of Dr. E. Parmly,

*Resolved,* That no address or essay shall occupy more than a half hour.

On motion of Dr. Westcott,

*Resolved,* That one day of the next session shall be set apart, and exclusively devoted to rehearsal of practice, and general discussion; questions upon practical subjects, and oral answers by any who may be disposed to give them.

Adjourned to the first Tuesday in August, 1846.

AMOS WESTCOTT, *Rec. Sec'ry.*

