# Correction

**Published:** 1845-12

**Authors:** Amos Westcott

**Affiliations:** Rec. Sec'y.


*Correction.*—The record of the proceedings of the last meeting, in regard to Dr. S. P. Hullihen, relative to the publication of Dr. Baker's Essay, was incomplete, and as left, calculated to give an erroneous, and unjust impression of the latter.

The call to order, was made by myself, and was made on the supposition that Dr. H. was not speaking to a motion. It appearing, however, that his remarks were explanatory to a motion, which I had failed to understand, the call was cheerfully withdrawn, and the society requested him to proceed—considering his remarks for such a purpose, of course, in order.

AMOS WESTCOTT, *Rec. Sec'y.*

